# Perceived Risk of Diabetes Among Vietnamese Americans With Prediabetes: Mixed Methods Study

**DOI:** 10.2196/39195

**Published:** 2023-04-14

**Authors:** Angelina Nguyen, Marylyn Morris McEwen, Lois J Loescher

**Affiliations:** 1 Louise Herrington School of Nursing Baylor University Dallas, TX United States; 2 College of Nursing The University of Arizona Tucson, AZ United States

**Keywords:** risk perception, perceived risk, diabetes, prediabetes, Vietnamese, Asian Americans

## Abstract

**Background:**

Vietnamese Americans have a relatively high risk of developing diabetes at younger ages, yet there are no published studies exploring their risk perceptions.

**Objective:**

This mixed methods study describes perceived diabetes risk in the context of an underserved population.

**Methods:**

This study was guided by the Common-Sense Model of Self-Regulation. Snowball sampling was used to recruit 10 Vietnamese Americans with prediabetes and achieve data saturation. Qualitative and quantitative descriptive methodologies with data transformation were used to analyze data from semistructured interviews and questionnaires to explore the dimensions of perceived diabetes risk.

**Results:**

Participants were between the ages of 30 and 75 years with diversity also noted in diabetes risk factors. The 3 risk perception domains from qualitative data were risk factors, disease severity, and preventing diabetes. The main perceived diabetes risk factors were eating habits (including cultural influences), sedentary lifestyle, and family history of diabetes. Quantitative data supported qualitative findings of a low-to-moderate level of perceived diabetes risk. Despite the lower levels of perceived diabetes risk, Vietnamese Americans do believe that the severity of diabetes is a “big concern.”

**Conclusions:**

Vietnamese Americans with prediabetes have a low-to-moderate level of perceived diabetes risk. Understanding the perceived diabetes risk in this population provides a foundation for diabetes prevention interventions that consider cultural influences on diet and exercise.

## Introduction

### Background

A diagnosis of prediabetes increases one’s risk of developing type 2 diabetes mellitus (T2DM) [1]. Prediabetes has been associated with multiple other chronic conditions including cardiovascular disease, chronic kidney disease, cancer, and dementia [1]. Despite having a BMI within normal limits, Vietnamese Americans experience a disproportionate burden of diabetes with higher diabetes prevalence rates compared with non-Hispanic Whites, having 60% higher odds of diabetes (*P*=.03) [2]. The average age of initial T2DM diagnosis in Vietnamese Americans is more than 5 years younger than that in non-Hispanic Whites [3]. Despite these increased risk factors, there are no publications for diabetes prevention studies targeted at the Vietnamese American population.

Exploring perceived T2DM risk in Vietnamese Americans with prediabetes provides a foundation for developing effective culturally appropriate strategies to alter risk perception that facilitates adoption and sustainment of T2DM prevention behaviors. Risk perception includes the cognitive and affective dimensions related to perspectives of general and personal risk [4]. Perceived T2DM risk has been positively associated with behavioral intention and perceived behavioral control; behavioral intention is associated with the likelihood of adopting preventive behaviors [5]. The purpose of this study was to describe the perceived risk of developing T2DM among Vietnamese Americans adults with prediabetes using a mixed methods approach. The specific aims were to (1) explore the domains of perceived risk of developing T2DM; (2) measure the level of perceived risk; and (3) synthesize transformed qualitative and quantitative data to describe this population’s perceived risk of developing T2DM.

### Conceptual Framework

**Figure 1:**
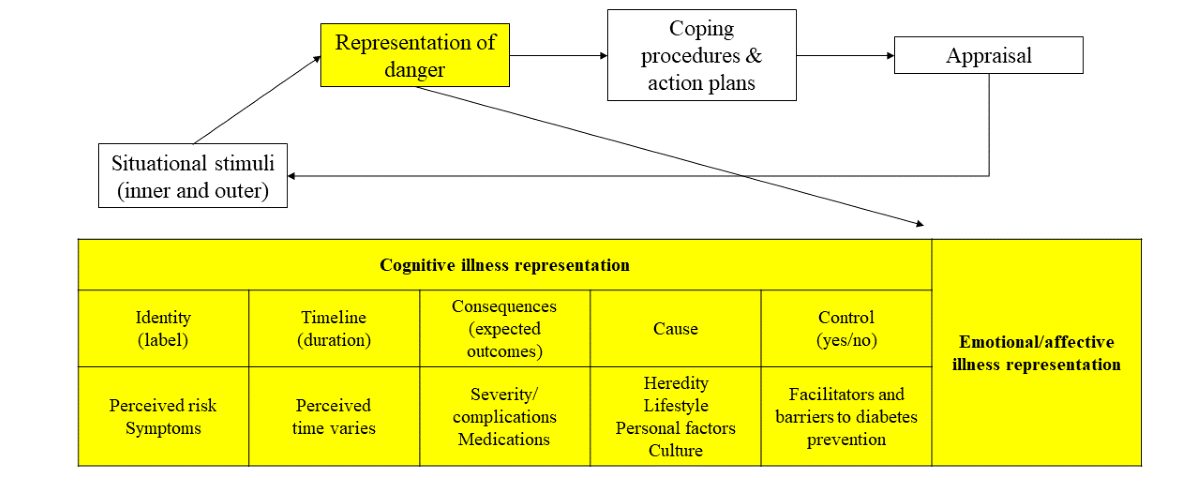
The Common-Sense Model of type 2 diabetes in Vietnamese Americans.

The Common-Sense Model (CSM) of Self-Regulation (Figure 1) is a process-oriented model that begins with developing the individual’s representations of illness in response to some sort of stimuli (internal or external), and followed by the development, implementation, and appraisal of action plans or coping methods [6]. The CSM has been used as a theoretical framework in this study to explore the perceived risk of developing T2DM in Vietnamese Americans. Illness representation is a central concept of the CSM, with emotional and cognitive dimensions. The cognitive dimension can be defined descriptively by its 5 constructs: identity (label) or symptoms and names of the threat, timeline (duration or age of onset) of the threat, consequences (expected outcomes) of the threat, cause of the threat, and control or cure for the threat [6].

## Methods

### Study Design

**Figure 2:**
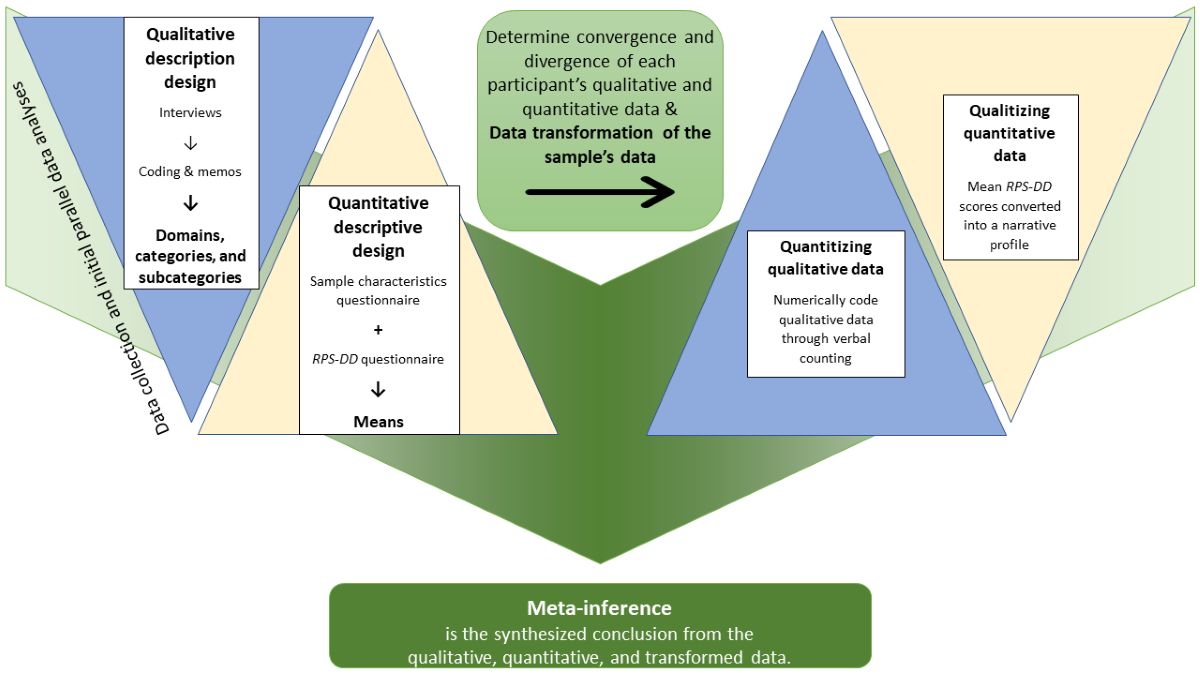
QUAL + quant mixed method research design and process. QUAL: qualitative; quant: quantitative; RPS-DD: Risk Perception Survey for Developing Diabetes.

A QUAL + quant mixed methods design (Figure 2) was used as follows: a qualitative description with semistructured interviews for aim 1, a quantitative descriptive design with questionnaires for aim 2, and the mixed method design–enabled generation of a meta-inference (synthesized analyses) from both qualitative and quantitative data for aim 3. With the QUAL + quant mixed method design, the qualitative methodology was prioritized and both the qualitative and quantitative methods were conducted concurrently [7,8].

### Participants

Various religious and community gatekeepers (eg, a priest, a monk, and business owners) of the Vietnamese communities in the Southwestern United States distributed recruitment flyers. Inclusion criteria were self-reported and as follows: (1) Vietnamese ethnic descent, (2) prediabetes diagnosis, (3) age 18 years or older, and (4) English language proficiency. Reading and spoken English language proficiency was determined through conversation with the principal investigator (PI). Potential participants contacted the PI directly. Snowball sampling was used to reach this very specific population, where participants were asked to refer other potential participants [9]. The recommended sample size in this qualitative dominant study was dependent on the number of participants needed to achieve data saturation (ie, when no new categories emerge from the data) [9]. Guest et al [10] found that 6 interviews will typically reach 80% saturation. Participant compensation included a US $10 gift card with the option to enter a raffle for 1 US $50 gift card.

### Data Collection and Analysis

#### Qualitative Data

The PI collected data using individual, semistructured face-to-face interviews in English between August and December 2018. Trustworthiness of qualitative findings was maintained by considering transferability, credibility, dependability, and confirmability. The CSM constructs were used to create the interview guide (Multimedia Appendix 1). While the interview questions with phrasing were planned, there was some flexibility warranted in the order that the questions were asked and clarifying questions as needed. Atlas.ti (ATLAS.ti Scientific Software Development GmbH) was used to organize the verbatim interview transcriptions, coding, memos, and analyses. Other data sources included field notes recorded by the PI.

Qualitative content analysis involved an iterative process with ongoing, alternating, and simultaneous data collection and data analysis [11]. Qualitative content analysis involved deductive (based on the defined dimensions of risk perception) and inductive (open coding) approaches to code, analyze, and interpret the data [11]. Reading and rereading the interview transcripts with the use of in vivo (participants’ own words) coding when possible maintained credibility of qualitative analysis. The PI (AN) and a coauthor (MMM) conducted open coding of 3 interviews independently, comparing codes and reaching consensus for congruency; all transcripts and analyses were further reviewed during weekly debriefing meetings. The PI created a codebook of codes with descriptions, as well as exemplary quotes from which the codes were derived. The codes were first classified into subcategories. Comparing the codes from all participants and generating subcategories and categories from like codes aided in the classification of domains, which was reviewed by the PI and a second coauthor (LJL) in additional debriefing meetings. Debriefing meetings held with the research team further enhanced credibility of findings. For assurance of dependability and confirmability, member checking was performed through shared interview transcript and final report with 2 participants. Member checking allowed for verification of accurate representation of risk perception and assurance that data saturation had been achieved. Finally, an audit trail was conducted by the coauthors to ensure the consensus of coding/meanings, consistency of the analyses processes, and the product of inquiry.

#### Quantitative Data

A total of 2 questionnaires in English were used to obtain sample characteristics and level of perceived diabetes risk. A PI-developed questionnaire was used to obtain sample characteristics such as demographics (ie, age range, gender, educational level, income, insurance status, and marital status), acculturation level (ie, immigrant generational status), BMI (calculated from self-reported height and weight), and diabetes disease history (ie, length of time since first diagnosed with prediabetes, history of gestational diabetes, and family history of diabetes).

The 43-item *Risk Perception Survey for Developing Diabetes* (RPS-DD) was used to measure the level of perceived diabetes risk [12]. The RPS-DD is a reliable and valid measure of perceived diabetes risk (Cronbach α=.84) and is the most widely used survey [4,13]. The composite RPS-DD score was calculated using the average of only 32 items (scored using a 4-point Likert scale, with 1=low attribute and 4=high attribute; reverse scoring was performed for some items to conform with the conceptual direction of the composite score) and does not include scoring for 11 items that only measure diabetes risk knowledge. The 5 subscales for the risk perception dimensions were Personal Control, Worry, Optimistic Bias, Personal Disease Risk, and Comparative Environment Risk (subscale Cronbach α values ranged from .50 to .81) [13]. An average of each risk perception subscale score was calculated by allowing an indicator of low versus high perception of each subscale concept (eg, high vs low perception of Personal Control). Descriptive statistics were used to analyze the data from the background information (ie, mean and frequencies) and the RPS-DD scores (ie, mean and internal consistency). SPSS 25.0 (IBM, Inc) was used to run all quantitative analyses.

### Meta-Inference: Synthesis of Qualitative and Quantitative Data

Meta-inferences were derived from the syntheses of the qualitative, quantitative, and transformed results [7]. This process began with determining data convergence and divergence. Convergence was determined if the qualitative and quantitative data for each participant were similar [8]. Divergence was determined if the qualitative and quantitative data for each participant were dissimilar [8].

Data transformation (ie, quantitizing qualitative data and qualitizing RPS-DD data) was also performed. The procedure for transforming data from qualitative to quantitative (and vice versa) was to (1) numerically code (quantitize) qualitative data through verbal counting and (2) convert quantitative data into a narrative profile [7]. The use of verbal counting confirmed the description of the patterns that have been found in the data [11]. The mean RPS-DD scores were transformed into a qualitative narrative profile pulled from exemplars of qualitative data [11]. The narrative profile was the basis for the meta-inferences of this mixed methods study.

### Ethics Approval

All study procedures were approved by the University of Arizona's institutional review board (protocol approval number: 1807760846). The University of Arizona maintains a Federal-wide Assurance with the Office for Human Research Protections (FWA #00004218).

## Results

### Sample Characteristics

A total of 10 participants were interviewed and completed questionnaires (Table 1). Half of the participants (n=5) were male. Most participants were married or had a domestic partner (n=6, 60%), had a household income greater than US $75,000 (n=6, 60%), and were first-generation immigrants (n=8, 80%). Ninety percent were overweight/obese. Regarding disease history, 50% (n=5) of the participants had a family history of diabetes, none had a history of gestational diabetes, and 80% (n=8) were initially diagnosed with prediabetes over a year ago.

**Table 1:** Sample characteristics (N=10).

Sample characteristics		Values, n (%)
Male		5 (50)
First-generation immigrant		8 (80)
Family history of diabetes		5 (50)
**Age (years)**		
	30-39	3 (30)
	40-49	2 (20)
	50-59	2 (20)
	≥60	3 (30)
**Initial prediabetes diagnosis (years)**		
	<1	2 (20)
	1-5	7 (70)
	>5	1 (10)
**Marital status**		
	Married/domestic partner	6 (60)
	Divorced/separated/widowed	2 (20)
	Never married/no domestic partner	2 (20)
**Household annual income (US $)**		
	<25,000	1 (10)
	25,000-49,999	1 (10)
	50,000-74,999	2 (20)
	>75,000	6 (60)
**Education level**		
	Some college	5 (50)
	Bachelor’s degree	3 (30)
	Postgraduate degree	2 (20)
**BMI category^a^**		
	Normal (<23 kg/m^2^)	1 (10)
	Overweight (23-24.9 kg/m^2^)	4 (40)
	Obese (≥25 kg/m^2^)	5 (50)
	Mean (SD)	27.70 (6.44)

^a^The BMI category was based on lowered BMI threshold (from 25 to 23 kg/m^2^) for T2DM screening in Asian Americans with overweight [14].

### Qualitative Results: Risk Perception Domains and Verbal Counting

#### Overview

The 3 risk perception domains emerged from the qualitative data: (1) perceived risk factors of prediabetes and diabetes (with categories for health behaviors and personal, health, and family history); (2) perceived disease severity with categories for prediabetes and diabetes; and (3) preventing T2DM with categories for behavior changes and the factors influencing those changes (Table 2). It should be noted that each of these domains are a component of the overall perceived diabetes risk. Participant names are pseudonyms to protect their identity.

**Table 2:** Domains, categories, and subcategories (including definitions) and related CSM^a^ constructs measured.

Domain, category, and subcategory				Definition of subcategory (CSM construct)
**Risk factors for prediabetes or diabetes**				
	**Health behaviors**			
		Sedentary lifestyle	Lack of exercise (cognitive dimension: cause)	
		Eating habits	Unhealthy eating habits (cognitive dimension: cause)	
		Stress management	Poor stress management (cognitive dimension: cause)	
	**Personal, health, and family history**			
		Personal factors	Characteristics of the individual that increases diabetes risk (cognitive dimension: cause)	
		Comes from family	Family history of diabetes (cognitive dimension: cause)	
		Cultural influences	Cultural influences include geographic and ethnic influences (cognitive dimension: cause)	
**Disease severity**				
	**Prediabetes diagnosis**			
		Initial reactions	Initial thoughts and feelings to being diagnosed with prediabetes (affective dimension)	
		Health concerns	Concerns regarding physical well-being (cognitive dimension: consequences)	
		I am at risk	Presence of perceived diabetes risk (cognitive dimension: identity)	
		Diabetes risk	Level of perceived diabetes risk (cognitive dimension: timeline)	
	**Diabetes**			
		Medication	Taking diabetes medication(s) (cognitive dimension: identity and consequences)	
		Complications	Diabetes complications (cognitive dimension: identity and consequences)	
**Preventing T2DM^b^**				
	**Behavior changes**			
		Modifying behaviors	Examples of health-promoting behavior changes (cognitive dimension: control and affective dimension)	
		Results of behavior changes	Results from health-promoting behavior changes (cognitive dimension: control and affective dimension)	
	**Factors influencing health behavior changes**			
		Become aware	Awareness of the need for health-promoting behavior changes (cognitive dimension: control and affective dimension)	
		I do the best I can	Personal efforts toward health promotion (cognitive dimension: control and affective dimension)	
		It hit home hard	Motivational influences on health promotion (cognitive dimension: control and affective dimension)	
		Barriers to preventing T2DM	Factors that impede health promotion (cognitive dimension: control and affective dimension)	

^a^CSM: Common-Sense Model.

^b^T2DM: type 2 diabetes mellitus.

#### Perceived Risk Factors of Prediabetes and Diabetes

This domain consisted of 2 categories: health behaviors and personal, health, and family factors. The health behaviors perceived by participants as risk factors were sedentary lifestyle, eating habits, and stress management. Other risk factor subcategories included personal factors (eg, increased age, race, and obesity), “coming from family” (ie, hereditary linkage), and cultural influences (eg, geographical, Vietnamese, and American influences). The risk factors demonstrating the greatest pattern were eating habits and cultural influences, supported by the higher frequency of these subcategories (n=9 for eating habits and n=8 from cultural influences) presented in the sample (Multimedia Appendix 2).

Most participants talked about eating large amounts of carbohydrates (including food and beverages high in sugar), sodium-rich foods and fish sauce, and fatty foods. Several participants mentioned that the main source of carbohydrates was rice and rice products. When considering influences on dietary habits, participant John (male, 36 years old) talked about how overeating and unhealthy food choices were engrained early in life and these habits were difficult to break. Participants Chinh (male, 44 years old), Huy (male, 51 years old), and Lan (female, 67 years old) described the social influences on their eating. Chinh referred to how he now limits socializing with his friends because of the associated drinking of alcohol which he believes leads to excessive eating. Huy says that when “my friends coming to town, or my poor health father, or my mom, and we decide to go for *pho* [Vietnamese rice noodle soup]...I will eat that pho.” Lan talked about how she makes healthier food choices when she eats alone, but that most of her meals are with her family and her husband (who cooks the meals) will get upset if she does not eat those foods.

The main cultural influence discussed was the Vietnamese culture, with most of the discussion focused on those cultural influences for unhealthy diets. The participants reiterated how Vietnamese “eat a lot of rice-based dishes.” Participant Jane (female, 30 years old) mentioned how Vietnamese “love their tropical fruit and that’s probably packed with sugar like jackfruit and...lychee definitely.” Jane also talked about traditional foods served during holidays such as *banh chung* and *banh tet* (Vietnamese dishes made primarily of glutinous sweet rice and mung bean) which are “in your childhood and it comes up as tradition...and takes you back to that feel-good moment.” While participants Chinh, Mai (female, 39 years old), and Quan (male, 44 years old) all denied a cultural influence on their risk, Quan later recanted when he talked about how Vietnamese “get arguably the worst of both worlds [Vietnamese and American] because you get more holidays...[which means] more of the celebratory meals.”

Other than the Vietnamese cultural influence, participant Huy talked about the regional influence of living in Las Vegas. He described how “you get free coupon, two-for-one, five-for-one, for seniors [at the buffet].” He went on to say that many Vietnamese “are gamblers [or casino dealers], so they have tons of comps [free compensated meals given by the casinos]...so they bring their friends and their family...”

#### Disease Severity

This domain consisted of 2 categories: prediabetes diagnosis and diabetes. The prediabetes diagnosis category refers only to the severity of being prediabetic and their risk for developing diabetes. The 4 subcategories for prediabetes diagnosis were their initial reactions to their prediabetes diagnosis, health concerns related to prediabetes, “I am at risk,” and diabetes risk. The diabetes category for the disease severity domain refers to their perceived risk of having developed diabetes; the 2 subcategories were medication and complications.

The subcategory with the highest frequency of participant reporting (n=10, 100%; Multimedia Appendix 2) and depth of answers was the initial reactions to the prediabetes diagnosis, which ranged from “not worried,” questioning the diagnosis, “slightly surprised,” “caught me off-guard,” “frightened,” and “freaked out.” Participant Yen (female, 72 years old) was not worried by her diagnosis saying that she is “too old right now...and can live around 10 more years.” Participant Jane mentioned that she “was just glad that it was prediabetes and not actual diabetes” as she notes not being very surprised by the diagnosis given her family history of diabetes. Participant Chinh instead reflected on his initial diagnosis of prediabetes by the health care provider stating how shocked he was, how he began questioning the diagnosis, and even stating that he may have overreacted to the diagnosis:

In my head, he’s givin’ me all this bad news, and I think I kinda tuned out a lot of things that he was saying. I was thinkin’, ‘how did I get this far?’ Because I was at that state. To me, it just sounded worse when I was sitting there.

Finally, participants Mai and Quan mentioned being “frightened” or “a little bit freaked out” as they considered their current experiences or knowledge regarding diabetes related to the need for medications (eg, insulin) or the complications of diabetes (eg, amputations and death). As participants continued to talk about the severity of diabetes (ie, the second category of disease severity), many noted that developing diabetes would be a “big concern” as evidenced by most participants reporting that medications will be needed (n=7) or complications may arise (n=6) if they develop T2DM.

While there was a wide range of how participants responded to their diagnosis of prediabetes, 9 participants further discussed either their perceptions of having risk for diabetes (n=8) or their level of diabetes risk (n=6). Overall, the participants who described their diabetes risk as low gave the following reasons: (1) they perceived that by developing a healthier lifestyle (ie, “eat better” and “exercise more”) they are almost eliminating their diabetes risk and (2) the risk is never nonexistent. They also perceived that they have a lower diabetes risk than the general population by citing the increased rates of obesity among Americans. Therefore, while participant Yen stated at one point in the interview that she did not think that she was at risk for developing diabetes, she does know that she cannot eliminate all risk.

#### Preventing T2DM

The final domain labeled “preventing T2DM” provided data regarding both the affective and cognitive dimensions of the CSM. This domain consisted of 2 categories: health behavior changes and factors influencing health behavior changes. The factors influencing health behavior changes were subcategorized to become aware, “I do the best I can,” “it hit home hard,” and barriers to preventing diabetes. Half of the participants noted the moment they were diagnosed with prediabetes by their health care provider that sparked the awareness needed to make the recommended lifestyle changes—whether it be the needed dietary modifications, increase in physical activity, or improvement of diabetes-related distress.

### Quantitative Results: RPS-DD Scores

The mean composite RPS-DD score was 2.15, indicating an overall low-to-moderate perceived risk of developing diabetes (Table 3). Participants had moderate-to-high levels of perceived Personal Control (mean score 3.30), Worry (mean score 3.10), and Optimistic Bias (mean score 2.75; this lower score indicates a higher level of comparative personal risk). Participants had low-to-moderate levels of perceived comparative Personal Disease Risk (mean score 2.06) and perceived Comparative Environmental Risk (mean score 2.27), relating to their perceptions of personal risk of diabetes, diabetes-related health complications, other diseases, and potential environmental hazards.

**Table 3:** RPS-DD^a^ results.

RPS-DD	Reliability (Cronbach α)	Range of scores	Mean scores (SD)
Composite RPS-DD^b^	.64	1.47-2.59	2.15 (0.31)
Personal Control Subscale^c^	.65	2.25-4.00	3.30 (0.59)
Worry Subscale^c^	.83	2.00-4.00	3.10 (0.81)
Optimistic Bias Subscale^d^	.70	1.50-4.00	2.75 (0.89)
Personal Disease Risk Subscale^e^	.52	1.53-2.80	2.06 (0.40)
Comparative Environmental Risk Subscale^c^	.86	1.00-3.44	2.27 (0.78)
Knowledge, %	N/A	36.4-81.8	60.9 (14.9)

^a^RPS-DD: Risk Perception Survey for Developing Diabetes.

^b^The composite RPS-DD score is an average of the 5 subscales with reversed scoring of 4 items to conform with the conceptual direction of the composite score; composite scores range from 1 (low overall perceived diabetes risk) to 4.47 (high overall perceived diabetes risk) and the median score is 2.23, which indicates a moderate level of overall perceived diabetes risk.

^c^1=low perceived Personal Control/Worry/Comparative Environmental Risk and 4=high perceived Personal Control/Worry/Comparative Environmental Risk and the median score is 2.5, which indicates a moderate level of the corresponding subscale construct.

^d^Subscale is labeled as the measurement of Optimistic Bias but the 2 items of this subscale measure comparative risk with 1 (strongly agree that the perceived risk for T2DM and serious disease is decreased compared with other people with the same age and gender) and 4 (strongly disagree that the perceived risk for T2DM and serious disease is decreased compared with other people with the same age and gender). The median score is 2.5, which indicates a moderate level of Optimistic Bias.

^e^Subscale score ranges from 1 (low perceived comparative Personal Disease Risk) to 5 (high perceived comparative Personal Disease Risk; ie, Likert scale scores of 1-4 indicate perceived risk of a disease or health problem, where 1=low perceived risk and 4=high perceived risk, and adding 1 if there is a personal or family history); the median score is 3, which indicates a moderate level of this subscale construct.

### Meta-Inference: Narrative Profile

The meta-inference is best described via the narrative profile that was developed based on the 9 participants with a low-to-moderate mean RPS-DD composite score (Table 4). This profile presented an overall low-to-moderate level of perceived diabetes risk. A closer look at various dimensions of perceived risk revealed moderate-to-high levels of Personal Control and Worry, but low-to-moderate levels of Comparative Risk, Personal Disease Risk, and Comparative Environmental Risk.

**Table 4:** Descriptive profile for low perceived diabetes risk scores on the RPS-DD^a^ (N=9).

Level of mean scores for the RPS-DD subscales	Mean scores (SD)	Exemplary qualitative data^b^
Moderate-to-high Personal Control	3.31 (0.62)	*I would say [there is a 0 to 10% chance of my developing diabetes] because I’m gonna try to definitely develop healthier lifestyle, and eat better, and exercise more.* [Quan]
Moderate-to-high level of Worry	3.06 (0.85)	*I know that there are people that are diabetic that have to be amputated, which [is]...a big concern. There's also people that I know that die early because of that.* [John]
Low-to-moderate level of Comparative Risk^c^	2.89 (0.82)	*[I think others have a higher risk of diabetes than me because I think of] Americans as just being obese, or overweight, or not having a healthy lifestyle.* [Chinh]
Low-to-moderate level of Personal Disease Risk	2.03 (0.41)	*Well, the reason why is I say my risk [for developing diabetes] is low is because I don’t think anybody’s risk is nonexistent.* [Chinh]
Low-to-moderate level of Comparative Environmental Risk	2.19 (0.78)	*Just like you driving here today on the freeway, knowing the risk [of getting into a car accident].* [Huy]

^a^RPS-DD: Risk Perception Survey for Developing Diabetes.

^b^Names are pseudonyms.

^c^Subscale is labeled as measurement of Optimistic Bias but the 2 items of this subscale actually measure comparative risk with a higher score indicating a decreased perceived risk for diabetes and serious disease compared with other people with the same age and gender.

The narrative profile was a summation of qualitative and quantitative data from 9 participants. The researchers determined that convergence resulted from similarities between the qualitative and quantitative data of the first participants (n=8). Pulling together all of the qualitative domains, categories, and subcategories, these participants were determined to have low levels of perceived diabetes risk based on statements fitting at least one of these 3 categories: (1) explicit statements that they believed they had a low diabetes risk, (2) they perceived high levels of control over their diabetes risk, or (3) they believed that they had lower diabetes risk compared with the general population.

The final participant included in the narrative profile had divergence noted between the qualitative and quantitative data. Huy’s qualitative data indicated his awareness and perception of a high diabetes risk, but the quantitative data indicated a low perceived diabetes risk. Huy stated how much he hated the term “prediabetes” as he equated it to the idiosyncrasy of using a term such as prepregnant because “either you’re pregnant or you’re not pregnant.” Huy also stated that having prediabetes indicates that “60 to 70 percent of your beta cells have already been gone...which means [that he is] at risk to be a frank diabetic.” Possible explanations for divergence are detailed in the “Discussion” section.

Only 1 case was omitted from the narrative profile. For Anh’s qualitative data, the level of perceived risk fluctuated throughout the interview due to his uncertainty with recent prediabetes diagnosis. The quantitative data indicated a higher level of perceived diabetes risk. This omission was not deemed to weaken the meta-inference.

## Discussion

### Principal Findings

There have been no prior publications that explored risk perceptions of developing diabetes in Vietnamese Americans. The key finding of this study is the meta-inference that Vietnamese Americans with prediabetes have an overall low perception of diabetes risk. This meta-inference was drawn from data categorized into the following qualitative domains: perceived risk factors, perceived disease severity, preventing T2DM, and mean RPS-DD scores of 9 participants. The participant with uncertain qualitative data was omitted from the narrative profile. This participant had just been diagnosed with prediabetes the week prior and her follow-up appointment was still pending at the time of the interview. She was very uncertain as to what this diagnosis meant and was not able to form much cognitive or affective representations of her diagnosis.

As for the case of divergence, participant Anh was unique because of his medical background. In his qualitative data, he is very clear regarding his high risk for developing diabetes. Yet, his quantitative data indicated that he had a low perceived diabetes risk. His composite RPS-DD score was 1.5. This is likely because he has a great understanding on how to control diabetes once it develops.

### Perceived Diabetes Risk Compared With Actual Diabetes Risk

The participants from this study reported an overall perception of low diabetes risk, despite their diagnosis of prediabetes putting them at an increased risk for developing T2DM. Perceived personal risk is not always congruent with actual personal risk, and this was demonstrated in a study in which more than 78% of participants with elevated or high actual diabetes risk reported absent or slightly perceived diabetes risk [15]. The finding from our study was not surprising when compared with Heidemann et al’s report [15] of incongruency between perceived and actual risk. It was, however, surprising when considering that another study has shown that increased diabetes risk perception is associated with the Asian race (odds ratio 1.475; *P*<.001) [16]. The finding from this study emphasizes the need for analysis of the different Asian ethnic subgroups.

### Diabetes Risk Factors in Vietnamese Americans

The participants reported the following perceived risk factors: eating habits, sedentary lifestyle, stress management, personal factors (eg, being overweight or older), heredity, and various cultural influences. The perceived risk factors of prediabetes and diabetes that emerged from this study were similar to the top 3 perceived causes of T2DM (diet, heredity, and stress) identified in an ethnography of Vietnamese Americans diagnosed with diabetes [17]. The participants of this study reported risk factors according to the American Diabetes Association. The reported American Diabetes Association risk factors for T2DM are being overweight, increased age, having a family history of diabetes, and having had gestational diabetes [14]. Gestational diabetes was the only factor not reported in this study.

Overall, there was congruency regarding what the participants of this study perceived to be risk factors of developing diabetes with what is known to be a T2DM risk factor. That is, they did not report any perceptions regarding risk factors that differed from professional health knowledge. For example, “heat” was not mentioned as the cultural perceived cause of diabetes, as mentioned in an ethnographic study of Vietnamese Americans with diabetes (*P* 309) [18]. The lack of this finding was surprising as the concepts of *am* and *duong* are part of a traditional belief that hot versus cold elements are metaphysical causes of illness [18] and the majority of participants in this study were first-generation immigrants. Perhaps a reason for this difference is that the participants of this study had high levels of education and all speak English, while more than half of the participants of the Mull et al’s [19] study spoke little to no English.

### Implications for Diabetes Prevention in Vietnamese Americans

Based on how many participants in this study noted their initial diagnosis as a motivator for adopting recommended lifestyle changes, there is a need for increased diabetes screening among Vietnamese Americans. Increased diabetes screening in this population is supported when considering that nearly one-half of Asian Americans with diabetes are undiagnosed [14]. The screening recommendations by the American Diabetes Association also include using more stringent diabetes screening criteria in Asian Americans with a lowered BMI threshold (from 25 to 23 kg/m^2^) for T2DM screening in Asian Americans with overweight [14].

For participants in this study, being overweight was overshadowed by the perceived risk of having a sedentary lifestyle. For example, participant John reported that his problems with prediabetes started when his lifestyle changed, and he was no longer active. He goes on to say that this sedentary lifestyle led to his weight gain. Sedentary lifestyle was also mentioned in reference to the perceived cultural influences on diabetes risk (eg, participant Lan’s perception that Vietnamese people simply do not exercise), clearly indicating the need to encourage physical activity in Vietnamese Americans.

Looking more closely at the perceived risk factors domain from this study, the most predominant subcategory was eating habits (n=9). The reported high carbohydrate and sodium-rich diet presents targets when developing interventions for Vietnamese Americans to improve their eating habits. Balanced diet and moderation may help to lower T2DM risk [20]. While the MyPlate nutrition guide by the US Department of Agriculture recommends that fruits comprise approximately 10% of the daily diet, there is no evidence that Vietnamese Americans who “love their tropical fruit” have an increased risk of T2DM due to the types of fruits that eat despite the higher glycemic indices of many tropical fruits [21]. So again, moderation is the key to avoid a higher total glycemic load associated with an increased risk of T2DM.

Sandelowski [11] stated that the use of verbal counting in qualitative research is useful for pattern recognition. Therefore, the increased frequency of data supporting the eating habits subcategory provides a focus for future diabetes prevention interventions with Vietnamese Americans. The cultural adaption of diabetes prevention programs for Asian American populations is necessary to improve program relevance, satisfaction, and participation [22], as well as to promote healthy behavior changes to reduce T2DM risk. The need for emphasis on interventions focused on dietary control is supported by 1 study participant who stated that Vietnamese Americans “need someone to show us a diet to follow” (*P* 78) [23]. Furthermore, nutrition has been the main focus of diabetes self-management among Vietnamese [23]. The findings of eating habits combined with the awareness of the cultural influences on eating habits may inform future culturally tailored diabetes prevention efforts.

Stress management is a strategy used in diabetes prevention programs [22]. Stress was mentioned by participants Jane, Anh (female, 50 years old), and Yen. As Jane explained this, “stress breaks down a body...it could cause you to have diabetes...if you don’t feel stressed, your heart feels lighter...and you don’t reach out for those comfort foods.” While the effects of stress on diabetes risk are not limited to eating behaviors, the emotion-oriented coping mechanism is certainly a consideration for interventions aimed at stress management in persons with or at risk for diabetes [21].

While there was an overall low perceived diabetes risk in this sample, they had a moderately high level of Personal Control. One previous study reported a positive correlation with a medium effect between the likelihood of adopting preventive behaviors and perceived behavioral control (*r*=0.308; *P*≤.001) in a sample of African American participants [5]. Given the associated increase in the likelihood of adopting health-promoting behaviors to prevent diabetes in the African American population, this may translate to Vietnamese Americans who also have a high level of Personal Control and therefore may be more amenable to lifestyle intervention programs.

The findings from this study create several focal points for diabetes prevention efforts aimed at the Vietnamese American population. Specifically, clinicians and researchers need to promote screening, encourage physical activity, and promote healthy eating. They also need to consider the barriers to preventing diabetes as reported by the participants in this study that included “limited time and feeling tired”; “convenient foods are not healthy”; “eating healthy is expensive”; “difficulty breaking unhealthy habits or mindsets”; “resisting cravings/temptations or lack of self-discipline”; “measuring quality of life by nonphysical attributes” (eg, the perception that eating something that is known to be unhealthy is more important because it means they are spending quality time with friends and family); and “not recognizing the importance of needed changes due to increased age and life expectancy.” Digital behavior change interventions should be used with (1) wider considerations around the user interface and design of content to promote accessibility and inclusivity and (2) novel automated approaches such as just-in-time approach to improve scalability [24,25].

### Limitations

The mixed method design was needed to explore the perceived risk of developing diabetes in Vietnamese Americans, as the sole use of qualitative versus quantitative designs would not have sufficiently revealed the influences of the Vietnamese and American cultures on eating habits or the level of perceived diabetes risk. The analyses for convergence and divergence of data, the verbal counting of qualitative subcategories, and the rich descriptions added to quantitative data from the interviews in the narrative profile increased the current understanding of perceived diabetes risk in Vietnamese Americans through elaboration [7,8]. However, the quantitative data analysis should be regarded cautiously, given the small sample.

With the small sample and required inclusion criterion of English proficiency, there is a limitation to the transferability and generalizability of the findings. The small sample size, in which saturation was achieved, was appropriate for this qualitative dominant design. Overall, the sample was diverse in terms of age, gender, marital status, and family history for diabetes. All participants in this study had some form of health care coverage, which is similar to other studies in which more than 90% of the Vietnamese American participants were insured through Medicare, Medicaid, or both [26,27].

### Conclusions

This study has important implications for nursing care and research as the Vietnamese American population experiences a disproportionate burden of diabetes, and there is a need to culturally tailor diabetes prevention programs to overcome this health disparity. This study provides culturally relevant data that inform future interventions targeted at modifiable risk factors. Based on the findings from this study, interventions that focus on healthy eating and the cultural influences for the adoption of this health-promoting behavior are essential. The use of the mixed methods research design allowed for greater understanding of risk perception than the sole use of either qualitative or quantitative methods, as well as greater capacity to inform both theory and practice. The awareness of high levels of Personal Control and Worry, as found in this sample, will help identify optimal candidates for diabetes prevention. Diabetes prevention efforts in Vietnamese Americans should also focus on increased screening for prediabetes and diabetes, as many participants reported an overall perception of low diabetes risk. Finally, it will also be important for health care providers to consider cultural influences when incorporating exercise into daily routines and for effective coping strategies in this population. As the preventing T2DM domain emerged from this study while the purpose was to uncover diabetes risk perception, a future study aimed at the current health behaviors and factors that influence those health behaviors could be further performed.

## Appendix

Multimedia Appendix 1 Main interview questions—basis for and resulting domains and categories.

Multimedia Appendix 2 Verbal counting for subcategories of qualitative data.

## Abbreviations

CSM: Common-Sense Model
RPS-DD: Risk Perception Survey for Developing Diabetes
T2DM: type 2 diabetes mellitus
