# Supplementary material for: Perceived Risk of Diabetes Among Vietnamese Americans With Prediabetes: Mixed Methods Study
Source: Asian Pac Isl Nurs J. 2023 Apr 14;7:e39195. doi: 10.2196/39195 (PMC10148206; doi:10.2196/39195)
Supplement: Multimedia Appendix 1 [file apinj_v7i1e39195_app1.docx]

*Interview Questions- Basis for and Resulting Domains and Categories*

| Basis from Common Sense Model | Interview Questions | Predominant Domain and Category from Data |
| --- | --- | --- |
| Information influencing risk perception & initial cognitive and emotional perspectives | What were your thoughts and/or feelings when you were first told by your health care provider that you had prediabetes? | Disease Severity: Prediabetes Diagnosis |
| Cause | What is your understanding of prediabetes? | Disease Severity: Prediabetes Diagnosis |
| Identity & Timeline & Consequences | What is your understanding of diabetes? | Disease Severity: Diabetes |
| Timeline | Now that you know you have prediabetes, how likely do you think it is that you will develop diabetes? | Disease Severity: Prediabetes Diagnosis |
| Cause | What are some of the factors that could put you at risk for developing diabetes? | Risk Factors: Health Behaviors and Personal, Health, and Family History |
| Control | What are some of the ways that you think you can prevent yourself from developing diabetes? | Preventing T2DM: Behavioral Changes |
| Cultural information influencing risk perception | How does being Vietnamese impact your perception of risk for developing diabetes in the future? | Risk Factors: Personal, Health, and Family History |
| All | Is there anything else you would like to tell me about what you believe to be your risk of developing diabetes in the future? | Risk Factors and Preventing T2DM Domains |

*Note.* T2DM= type 2 diabetes mellitus
