# Supplementary figures and images for: Perceived Risk of Diabetes Among Vietnamese Americans With Prediabetes: Mixed Methods Study
Source: Asian Pac Isl Nurs J. 2023 Apr 14;7:e39195. doi: 10.2196/39195 (PMC10148206; doi:10.2196/39195)

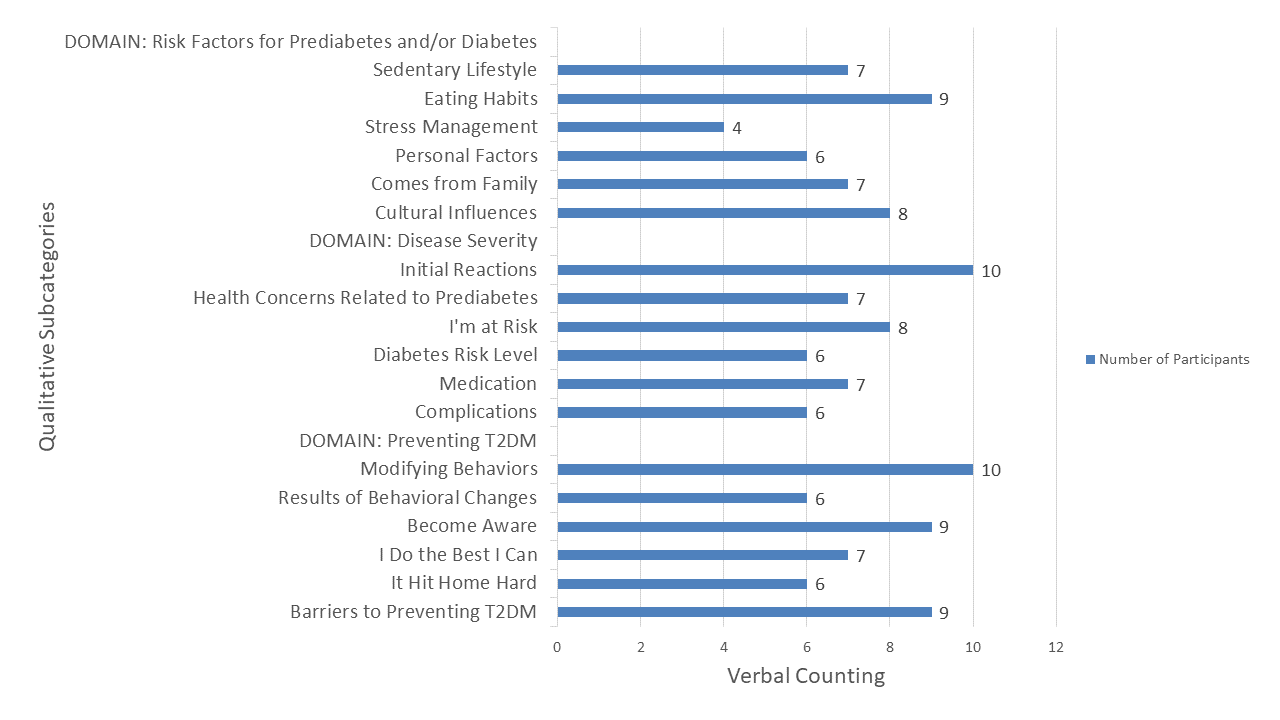

Supplement: Multimedia Appendix 2 [file apinj_v7i1e39195_app2.png]
